# Simultaneous Thermal Stability and Ultrahigh Sensitivity of Heterojunction SERS Substrates

**DOI:** 10.3390/nano9060830

**Published:** 2019-05-31

**Authors:** Lingwei Ma, Jinke Wang, Hanchen Huang, Zhengjun Zhang, Xiaogang Li, Yi Fan

**Affiliations:** 1 Institute of Advanced Materials & Technology, University of Science and Technology Beijing, Beijing 100083, China; mlw1215@ustb.edu.cn (L.M.); wjkgege@126.com (J.W.); lixiaogang@ustb.edu.cn (X.L.); 2 College of Engineering, University of North Texas, Denton, TX 76207, USA; 3 Key Laboratory of Advanced Materials, School of Materials Science and Engineering, Tsinghua University, Beijing 100084, China; 4 Jiangsu Key Laboratory for Premium Steel Material, Nanjing Iron and Steel Co., Ltd., Nanjing 210035, China; fanyi@njsteel.com.cn

**Keywords:** surface-enhanced Raman scattering (SERS), glancing angle deposition (GLAD), heterojunctions, SERS sensitivity, thermal stability

## Abstract

This paper reports the design of Ag-Al_2_O_3_-Ag heterojunctions based on Ag nanorods (AgNRs) and their applications as thermally stable and ultrasensitive substrates of surface-enhanced Raman scattering (SERS). Specifically, an ultrathin Al_2_O_3_ capping layer of 10 nm on top of AgNRs serves to slow down the surface diffusion of Ag at high temperatures. Then, an additional Ag layer on top of the capping layer creates AgNRs-Al_2_O_3_-Ag heterojunctions, which lead to giant enhancement of electromagnetic fields within the Al_2_O_3_ gap regions that could boost the SERS enhancement. As a result of this design, the SERS substrates are thermally stable up to 200 °C, which has been increased by more than 100 °C compared with bare AgNRs, and their sensitivity is about 400% that of pure AgNRs. This easy yet effective capping approach offers a pathway to fabricate ultrasensitive, thermally stable and easily prepared SERS sensors, and to extend SERS applications for high-temperature detections, such as monitoring in situ the molecule reorientation process upon annealing. Such simultaneous achievement of thermal stability and SERS sensitivity represents a great advance in the design of SERS sensors and will inspire the fabrication of novel hetero-nanostructures.

## 1. Introduction

Surface-enhanced Raman scattering (SERS) is the foundation of a powerful spectroscopic technique for rapid and non-destructive determination of chemical [1], environmental [2,3] and biological [4] analytes at trace levels, even at the level of a single molecule [5]. To achieve high sensitivity of SERS, the substrates are typically nanoscale noble metals, such as Ag, Au, and Cu, that have superior plasmonic efficiency [6,7]. While the nanoscale dimension gives rise to high sensitivity, it also leads to thermal instability because nanostructures coarsen easily at elevated temperatures [8,9]. For example, Ag nanorods (AgNRs) array and Ag colloids coarsen so much that they fuse at a temperature as low as 50 °C [10,11], thus limiting their practical SERS applications, such as monitoring in situ the thermal crystallization and the catalysis process.

Aiming to preserve the high sensitivity and yet to achieve thermal stability of SERS substrates, we have recently proposed and demonstrated the capping of AgNRs using high-melting temperature Al_2_O_3_ [12]. In contrast to oxide coating that also improves thermal stability [13,14,15,16], the oxide capping minimizes the reduction of exposed metallic surfaces that provide SERS sensitivity. A capping layer of 10 nm Al_2_O_3_ gives an optimal combination of thermal stability up to 200 °C and slight reduction of sensitivity by 25% [12]. The improved thermal stability opens the door for SERS applications in high-temperature environments. However, high-temperature SERS sensing often involves the detection of monolayer molecular adsorption and interface interactions [17,18,19], and therefore requires ultrahigh sensitivity. While preserving the improved thermal stability, is it possible to increase the sensitivity by 100% or more?

**Figure 1:**
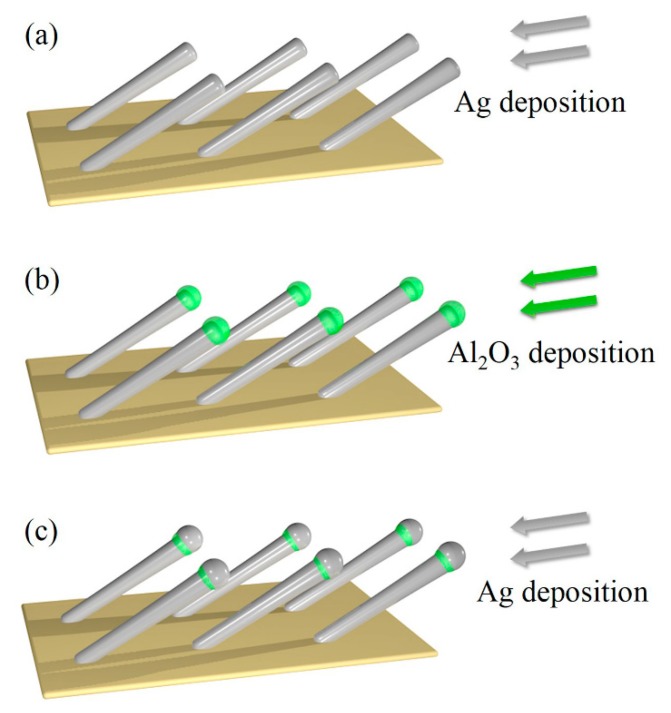
Schematic of (**a**) AgNRs deposition, (**b**) capping of AgNRs with Al_2_O_3_, (**c**) deposition of additional Ag.

To achieve this goal, we propose the design of Ag-Al_2_O_3_-Ag heterojunctions based on AgNRs. As conceptually illustrated in Figure 1, we first deposit AgNRs and cap them with Al_2_O_3_ layers, and then deposit additional Ag of optimal thickness. The AgNRs-Al_2_O_3_-Ag heterojunctions give rise to giant electromagnetic (EM) enhancement [20], which in turn leads to ultrahigh SERS sensitivity [21,22]. In this paper, we demonstrate the feasibility of this proposal using the glancing angle deposition (GLAD) technique [10,12]. Our experiments show that the proposed design leads to thermal stability up to 200 °C and SERS sensitivity up to 400% that of pure AgNRs. The SERS intensity of different AgNRs-Al_2_O_3_-Ag substrates first elevates with the Ag capping thickness, reaches a maximum at the Ag thickness of 150 nm, and declines beyond the critical thickness. The Raman signal of methylene blue (MB) is clearly measurable on the Ag-10Al_2_O_3_-150Ag substrate, even at a low concentration of 1 × 10^−10^ M. The SERS enhancement factor is on the order of 10^8^, which is comparable to the best reported results of AgNRs-based substrates [23,24,25], and the stable temperature of AgNRs-Al_2_O_3_-Ag increases by more than 100 °C. As a straightforward application of the heterojunctions, we exploit them to characterize the reorientation process of 4-tert-butylbenzylmercaptan (4-tBBM) molecules at elevated temperatures.

## 2. Materials and Methods

### 2.1. Fabrication and Thermal Annealing of AgNRs-Al_2_O_3_-Ag Substrates

AgNRs are deposited on Si (001) substrates using the GLAD technique in an electron-beam system with a background vacuum level down to 10^−5^ Pa. During deposition, the incident angle of the vapor flux is set at 86° off the substrate normal. The nominal deposition rate is fixed at 0.75 nm/s. The nominal rate refers to the rate with zero-degree incidence angle, as read by a quartz crystal microbalance (QCM). The total nominal deposition is 1000 nm in thickness. After the deposition of AgNRs, without breaking the vacuum, the target is switched to Al_2_O_3_ in the deposition chamber. The incidence angle is set at 86° and the deposition rate is 0.1 nm/s. Our choice of Al_2_O_3_ thickness is based on the following two considerations. First, Al_2_O_3_ thickness needs to be sufficiently small to avoid the excessive coverage of Ag surfaces. Second, it needs to be large enough to maximize the thermal stability. The detailed optimization process of Al_2_O_3_ thickness is discussed in the Supporting Information (see Figures S1–S4). On the one hand, the SERS efficiency of AgNRs-Al_2_O_3_ substrates with 6 nm to 12 nm capping declined moderately with Al_2_O_3_ deposition. On the other hand, by depositing 10 nm or thicker Al_2_O_3_ onto AgNRs, the substrates exhibited no discernible morphology variation at 200 °C. Therefore, the total nominal deposition thickness of Al_2_O_3_ we used in this work is 10 nm [12].

Finally, the evaporation target is switched to Ag again in the chamber, the incident angle is kept at 86°, and the deposition rate is decreased to 0.3 nm/s for better coverage of the Al_2_O_3_. The total nominal deposition of Ag is set as 20, 50, 100, 125, 150, 175 and 200 nm, respectively, for each test. The AgNRs-Al_2_O_3_-Ag substrates are annealed on a hot plate at each given temperature for 15 min in air. The morphology evolution process during annealing is monitored in situ via the reflectivity variations, using the Optical Power Thermal Analyzer (OPA-1200, Wuhan Joule Yacht Science & Technology Co., Ltd., Wuhan, China).

### 2.2. Characterizations of AgNRs-Al_2_O_3_-Ag Substrates – Morphology, Structure, and SERS 

The morphology and structure of the prepared SERS substrates are characterized using a scanning electron microscope (SEM, JEOL-JMS-7001F, Tokyo, Japan) and a high-resolution transmission electron microscope (HRTEM, JEOL-2011, Tokyo, Japan). SERS performance is characterized using an optical fiber micro-Raman system (i-Raman Plus, B&W TEK Inc., Newark, DE, United States), with MB and 4-tBBM as probing molecules. Raman spectra are collected based on an excitation laser of 785 nm in wavelength and of 100 mW in power, with a beam spot of ~80 microns in diameter. Before SERS characterizations, all substrates are immersed into analyte solutions for 30 min, washed thoroughly by deionized water to remove the residual molecules, and are then dried naturally in air. The data collection time for each spectrum is set to be five seconds and each SERS spectrum is obtained by measuring and averaging the signals collected from five different spots on a substrate.

### 2.3. FEM Simulation

Numerical simulations of AgNRs, AgNRs-Al_2_O_3_ and AgNRs-Al_2_O_3_-Ag were conducted using the finite element method (FEM) software COMSOL Multiphysics 5.2. Dimensional parameters were acquired from SEM observations, together with the excitation wavelength of 785 nm.

## 3. Results and Discussion

**Figure 2:**
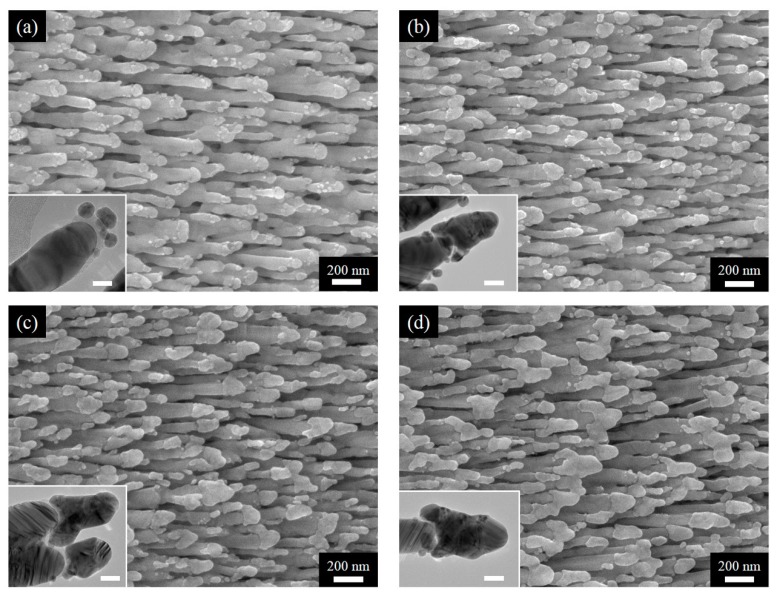
SEM images of AgNRs-Al_2_O_3_-Ag arrays with additional Ag deposition of (**a**) 50 nm, (**b**) 100 nm, (**c**) 150 nm, and (**d**) 200 nm; accompanying HRTEM images are included as insets with the scale bar being 20 nm.

As the first set of results, we present the SEM and HRTEM images of AgNRs-Al_2_O_3_-Ag arrays of various Ag deposition thickness on top of AgNRs-Al_2_O_3_. As shown in Figure 2, the additional deposition of 50 nm Ag leads to the formation of Ag nanoparticles. As the deposition thickness increases to 100 nm, these nanoparticles merge to cover the Al_2_O_3_ capping. The coverage is more complete as the thickness increases to 150 nm. Further increase of the thickness to 200 nm results in the bridging of neighboring nanorods. Therefore, moderate thickness of deposition, around 150 nm, leads to the heterojunctions that we conceptually proposed in Figure 1.

**Figure 3:**
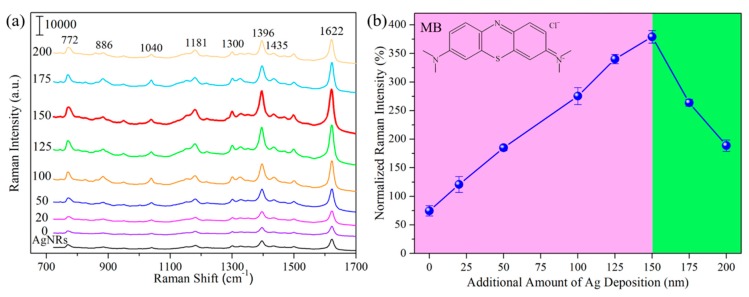
(**a**) SERS spectra of 1 × 10^−6^ M MB on bare AgNRs, and AgNRs-Al_2_O_3_-Ag arrays with additional Ag deposition of 0, 20, 50, 100, 125, 150, 175, and 200 nm; marked as AgNRs and the thickness of additional Ag deposition. (**b**) Raman intensity at 1622 cm^−1^, normalized with respect to that on bare AgNRs, as a function of the additional thickness of Ag deposition.

**Figure 4:**
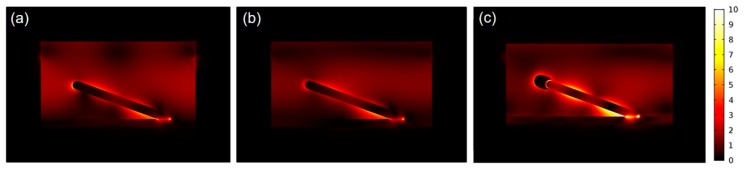
Localized electric field intensity distributions (indicated by the color bar) of (**a**) AgNRs, (**b**) AgNRs-10Al_2_O_3_; and (**c**) AgNRs-10Al_2_O_3_-150Ag, respectively.

Next, we examine the SERS sensitivity of AgNRs-Al_2_O_3_-Ag arrays. Figure 3a shows the SERS spectra of 1 × 10^−6^ M MB [26] on AgNRs-Al_2_O_3_-Ag arrays with various thickness of additional Ag deposition in the final stage. The spectrum on the AgNRs substrate is included for comparison. Characteristic Raman peaks are clearly seen in Figure 3a. In-plane bending of C–H is observed at 772 cm^−1^, 886 cm^−1^, and 1040 cm^−1^, and in-plane ring mode of C–H is at 1300 cm^−1^. 1181 cm^−1^ is assigned to the stretching of C–N. Besides, 1396 cm^−1^, 1435 cm^−1^ and 1622 cm^−1^ correspond to the symmetric and asymmetric C-N stretches, as well as the C-C ring stretching, respectively. Indeed, the additional deposition of Ag increases the sensitivity, and an optimal amount is around 150 nm, consistent with the heterojunction morphology that is shown in Figure 2. Taking the characteristic peak at 1622 cm^−1^ as reference, Figure 3b shows the Raman intensity, normalized by that on bare AgNRs, as a function of the additional thickness of Ag deposition. The SERS intensity of different AgNRs-Al_2_O_3_-Ag substrates first elevates with the Ag capping thickness, reaches a maximum at the Ag thickness of 150 nm, and declines beyond the critical thickness. This sensitivity variation trend can be explained by different Ag morphologies: As Ag thickness increases, Ag layers grow from small particles into complete capping over nanorod tips, which could generate stronger EM field in the gap regions. When the deposition thickness exceeds 150 nm, Ag capping coalesces into large aggregations without clear tips and gaps, in which way the SERS activity declines to some extent. Remarkably, with the optimal Ag deposition thickness of 150 nm, the intensity (and thereby sensitivity) is about 400% that on bare AgNRs. For better comparison, we prepared two reference substrates, i.e., AgNRs coated with 150 nm Ag (AgNRs-150Ag) and 10 nm Al_2_O_3_ capped with 150 nm Ag (10Al_2_O_3_-150Ag). As shown in Figure S5, without Al_2_O_3_ gap, the AgNRs-150Ag film looks similar to bare AgNRs, with a little increase in nanorod length; the 10Al_2_O_3_-150Ag substrate produces many nanoparticles on Si surface instead of nanorods. The SERS intensities on AgNRs-Al_2_O_3_-Ag arrays are much higher than those obtained on AgNRs-150Ag and 10Al_2_O_3_-150Ag, as well as their intensity sum. These results manifest that without Al_2_O_3_ gap or underneath AgNRs, the nanocomposite could not generate very strong SERS enhancement. As for the AgNRs-Al_2_O_3_-Ag substrate, the intense interaction between AgNRs and Ag capping layers gives rise to a huge EM field within the Al_2_O_3_ gap regions, which is crucial for SERS enhancement. Figure 4 presents the FEM modeling results of (a) AgNRs; (b) AgNRs-10Al_2_O_3_; and (c) AgNRs-10Al_2_O_3_-150Ag, respectively. AgNRs possess intense EM enhancement at the tip and side of nanorods, while the EM field at the tip of AgNRs-10Al_2_O_3_ decreases slightly after Al_2_O_3_ capping. After further Ag deposition of 150 nm, there are evident “hot spots” at the gap between the AgNRs and the Ag capping layer, leading to stronger EM enhancement at the tip and side of this heterojunction structure. This simulation result suggests that the AgNRs-10Al_2_O_3_-150Ag array is a superior platform for maximizing the SERS performance.

**Figure 5:**
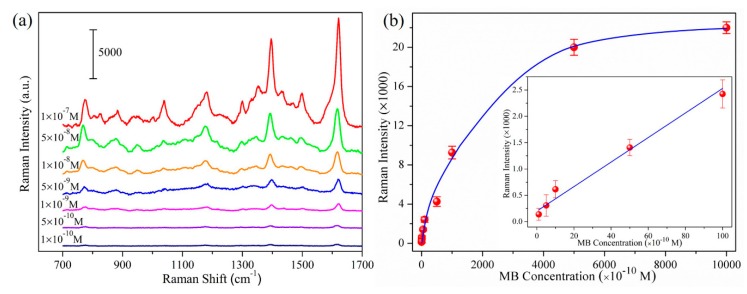
(**a**) SERS spectra of MB molecules on AgNRs-Al_2_O_3_-Ag substrates with 150 nm additional Ag; the thickness of MB molecules is marked by each spectrum; (**b**) Raman peak intensity at 1622 cm^−1^ as a function of MB concentrations, inserted: 1622 cm^−1^ peak intensity from 1 × 10^−10^ M to 1 × 10^−8^ M.

After discussing the optimization of the proposed substrates, we now examine the SERS efficiency and quantification capacity of the designed heterojunctions. Taking AgNRs-Al_2_O_3_-Ag arrays with 150 nm additional Ag as the substrate, Figure 5a shows the SERS spectra of MB molecules of various concentrations, and Figure 5b shows the Raman peak intensity at 1622 cm^−1^ as a function of the concentration from 1 × 10^−10^ M to 1 × 10^−6^ M. Even at the concentration of 1 × 10^−10^ M, the Raman signal is clearly measurable and follows the linear dependence on concentration. The peak intensity increases linearly with concentration from 1 × 10^−10^ M to 1 × 10^−8^ M. The limit of detection (LOD) of MB is about 9.3 × 10^−11^ M based on a signal-to-background ratio of S/N = 3. At higher concentrations, the intensity increases slowly due to the saturated adsorption. As described in the Supporting Information, the SERS enhancement factor reaches 1.3 × 10^8^, which is comparable to the best reported values of AgNRs-based substrates [23,24,25], reflecting the ultrahigh SERS efficiency of the AgNRs-Al_2_O_3_-Ag heterojunctions.

**Figure 6:**
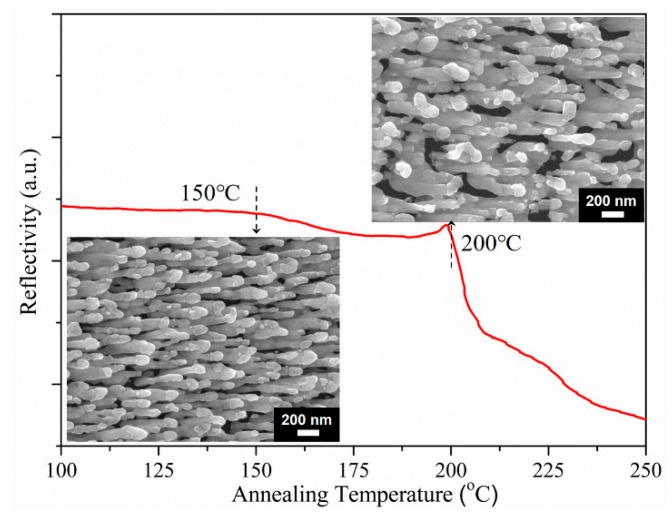
Reflectivity of the AgNRs-Al_2_O_3_-Ag arrays as a function of annealing temperature; the insets are SEM images of arrays after annealing at 150 °C (lower left) and 200 °C (upper right), respectively.

Having established the high sensitivity of AgNRs-Al_2_O_3_-Ag arrays with 150 nm additional Ag, we now examine their thermal stability upon annealing. Figure 6 shows the reflectivity of the AgNRs-Al_2_O_3_-Ag arrays as a function of the annealing temperature [16]. As expected, the Al_2_O_3_ capping layer still serves the purpose of slowing down the surface diffusion so as to increase the thermal stability up to 200 °C. In comparison with the morphology in Figure 2c, the annealing at 150 °C hardly changed the morphology and reflectivity. Although annealing at 200 °C leads to visible coarsening, the arrays remain separate and the reflectivity remains unchanged. As for bare AgNRs, they melted completely at 200 °C and experienced a substantial SERS enhancement degradation of ~90% once the temperature went beyond 100 °C [12]. Therefore, they were not feasible for high-temperature sensing.

**Figure 7:**
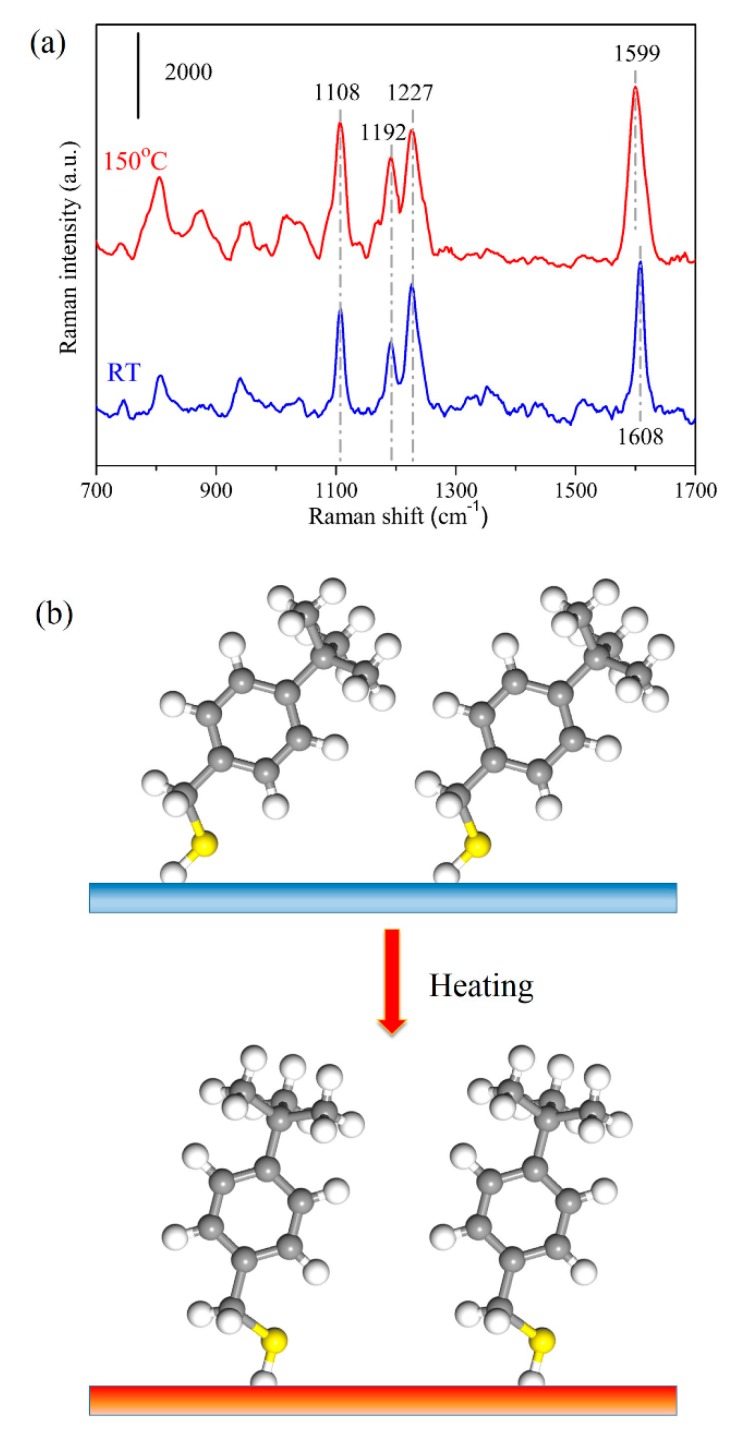
(**a**) SERS spectra of 1 × 10^−6^ M 4-tBBM molecules adsorbed on AgNRs-Al_2_O_3_-Ag arrays with 150 nm additional Ag deposition, at room temperature (RT) and upon heating at 150 °C for 5 min. (**b**) Schematic of 4-tBBM molecule reorientation process upon heating.

Finally, as an application, we investigate the reorientation process of 4-tBBM molecules on this unique AgNRs-Al_2_O_3_-Ag nanostructure. We first collected the SERS signals of 4-tBBM molecules on the substrate surface at room temperature, and then heated the analyte-adsorbed substrate at 150 °C for 5 min, followed by measuring the SERS spectra again. Figure 7a presents the SERS spectra of 4-tBBM adsorbed on AgNRs-Al_2_O_3_-Ag before and after annealing. At room temperature, there are prominent SERS peaks at 1108 and 1192 cm^−1^ (the vibration of the phenyl ring), 1227 cm^−1^ (the wagging of methylene groups) and 1608 cm^−1^ (8a mode) [27]. Upon heating to 150 °C, the SERS intensities at 1108 and 1192 cm^−1^ increased and the signal at 1227 cm^−1^ remained steady. Of particular interest is that the peak at 1608 cm^−1^ shifted to 1599 cm^−1^. These intensity and peak position variations imply that the orientation changes of 4-tBBM molecules on the substrate surface during heating. To be specific, the intensity increase at 1192 and 1108 cm^−1^ of in-plane modes of 4-tBBM suggests that the angle between the phenyl ring and the surface normal of the substrate decreases upon annealing [27], a schematic diagram of which is shown in Figure 7b. The peak shift from 1608 to 1599 cm^−1^ also verifies such a transition. Therefore, thermal energy accelerates the molecular vibration and drives 4-tBBM to rearrange to a more stable orientation. AgNRs-Al_2_O_3_-Ag thus holds great potential to determine the orientation and conformation of interfacial molecules on SERS substrates.

## 4. Conclusions

In conclusion, through the design of nanoscale heterojunctions, we have developed AgNRs-based SERS substrates that (1) have about 400% times the sensitivity of pure AgNRs and (2) are thermally stable up to 200 °C. The AgNRs-Al_2_O_3_-Ag heterojunctions are prepared facilely using physical vapor deposition under the glancing angle incidence. The optimal thickness of additional Ag deposition on top of AgNRs-Al_2_O_3_ is 150 nm. To demonstrate the impacts of the design, we have shown that the AgNRs-Al_2_O_3_-Ag arrays have an ultrahigh SERS enhancement factor on the order of 10^8^. Additionally, the heterojunction arrays allow the characterization of reorientation processes of interfacial 4-tBBM molecules on the substrate upon heating. The AgNRs-Al_2_O_3_-Ag arrays, with both superior SERS sensitivity and thermal stability, hold great potential for real-world SERS applications.

## Supplementary Materials

The following are available online at https://www.mdpi.com/2079-4991/9/6/830/s1, Figure S1: SEM images of (a) uncapped AgNRs and (b) AgNRs-10Al_2_O_3_ substrates; HRTEM images of AgNRs capped with Al_2_O_3_ layers of (c) 8, (d) 10 and (e) 20 nm, respectively. Figure S2: (a) SERS spectra of 1 × 10^−6^ M MB adsorbed on bare AgNRs and on AgNRs-Al_2_O_3_ substrates with different capping thickness; (b) The normalized intensities of 1622 cm-1 Raman peak of 1 × 10^−6^ M MB molecules as a function of the Al_2_O_3_ thickness of different AgNRs-Al_2_O_3_ substrates. Figure S3: SEM images of AgNRs and different AgNRs-Al_2_O_3_ substrates after annealing at 150 °C and 200 °C for 15 min. Figure S4: The reflectivity changes of different AgNRs-Al_2_O_3_ substrates upon annealing from 100 °C to 250 °C. Figure S5: SEM images of (a) AgNRs-150Ag and (b) 10Al_2_O_3_-150Ag substrates; (c) SERS spectra of 1 × 10^−6^ M MB molecules adsorbed on AgNRs-10Al_2_O_3_-150Ag, AgNRs-150Ag and 10Al_2_O_3_-150Ag substrates, separately.

## References

[1] Fateixa S., Nogueira H., Trindade T. (2015). Hybrid nanostructures for SERS: Materials development and chemical detection. Phys. Chem. Chem. Phys..

[2] Ma Y., Huang Z., Li S., Zhao C. (2019). Surface-enhanced Raman spectroscopy on self-assembled Au nanoparticles arrays for pesticides residues multiplex detection under complex environment. Nanomaterials.

[3] Sitjar J., Liao L., Lee H., Liu B., Fu W. (2019). SERS-active substrate with collective amplification design for trace analysis of pesticides. Nanomaterials.

[4] Kalachyova Y., Erzina M., Postnikov P., Svorcik V., Lyutakov O. (2018). Flexible SERS substrate for portable Raman analysis of biosamples. Appl. Surf. Sci..

[5] Chen H., Lin M., Wang C., Chang Y., Gwo S. (2015). Large-scale hot spot engineering for quantitative SERS at the single-molecule scale. J. Am. Chem. Soc..

[6] Liu K., Bai Y., Zhang L., Yang Z., Fan Q., Zheng H., Yin Y., Gao C. (2016). Porous Au-Ag nanospheres with high-density and highly accessible hotspots for SERS analysis. Nano Lett..

[7] Yang C., Zhang C., Huo Y., Jiang S., Qiu H., Xu Y., Li X., Man B. (2016). Shell-isolated graphene@Cu nanoparticles on graphene@Cu substrates for the application in SERS. Carbon.

[8] Bottani C., Bassi A., Tanner B., Stella A., Tognini P., Cheyssac P., Kofman R. (1999). Melting in metallic Sn nanoparticles studied by surface Brillouin scattering and synchrotron-x-ray diffraction. Phys. Rev. B.

[9] Pan C., Zhang Z., Su X., Zhao Y., Liu J. (2004). Characterization of Fe nanorods grown directly from submicron-sized iron grains by thermal evaporation. Phys. Rev. B.

[10] Bachenheimer L., Elliott P., Stagon S., Huang H. (2014). Enhanced thermal stability of Ag nanorods through capping. Appl. Phys. Lett..

[11] Mai F., Yang K., Liu Y., Hsu T. (2012). Improved stabilities on surface-enhanced Raman scattering-active Ag/Al_2_O_3_ films on substrates. Analyst.

[12] Ma L., Zhang Z., Huang H. (2017). Design of Ag nanorods for sensitivity and thermal stability of surface-enhanced Raman scattering. Nanotechnology.

[13] John J., Mahurin S., Dai S., Sepaniak M. (2009). Use of atomic layer deposition to improve the stability of silver substrates for in situ, high-temperature SERS measurements. J. Raman. Spectrosc..

[14] Mahurin S., John J., Sepaniak M., Dai S. (2011). A reusable surface-enhanced Raman scattering (SERS) substrate prepared by atomic layer deposition of alumina on a multi-layer gold and silver film. Appl. Spectrosc..

[15] Ma L., Huang Y., Hou M., Xie Z., Zhang Z. (2015). Ag nanorods coated with ultrathin TiO_2_ shells as stable and recyclable SERS substrates. Sci. Rep..

[16] Ma L., Wu H., Huang Y., Zou S., Li J., Zhang Z. (2016). High-performance real-time SERS detection with recyclable Ag nanorods@HfO_2_ substrates. ACS Appl. Mater. Interfaces.

[17] Masango S., Hackler R., Henry A., McAnally M., Schatz G., Stair P., Van Duyne R. (2016). Probing the chemistry of alumina atomic layer deposition using operando surface-enhanced Raman spectroscopy. J. Phys. Chem. C.

[18] Zhang J., Zhang D., Shen D. (2002). Orientation study of atactic poly(methyl methacrylate) thin film by SERS and RAIR spectra. Macromolecules.

[19] Formo E., Wu Z., Mahurin S., Dai S. (2011). In situ high temperature surface enhanced Raman spectroscopy for the study of interface phenomena: Probing a solid acid on alumina. J Phys. Chem. C.

[20] Willets K., Van Duyne R. (2007). Localized surface plasmon resonance spectroscopy and sensing. Annu. Rev. Phys. Chem..

[21] Lai Y., Chen S., Hayashi M. (2014). Mesostructured arrays of nanometer-spaced gold nanoparticles for ultrahigh number density of SERS hot spots. Adv. Funct. Mater..

[22] Shiohara A., Wang Y., Liz-Marzán L. (2014). Recent approaches toward creation of hot spots for SERS detection. J. Photochem. Photobiol. C.

[23] Han C., Yao Y., Wang W., Qu L., Bradley L., Sun S., Zhao Y. (2017). Rapid and sensitive detection of sodium saccharin in soft drinks by silver nanorod array SERS substrates. Sens. Actuators B Chem..

[24] Liu Y., Chu H., Zhao Y. (2010). Silver nanorod array substrates fabricated by oblique angle deposition: morphological, optical, and SERS characterizations. J. Phys. Chem. C.

[25] Driskell J., Shanmukh S., Liu Y., Chaney S., Tang X., Zhao Y., Dluhy R. (2008). The use of aligned silver nanorod arrays prepared by oblique angle deposition as surface enhanced Raman scattering substrates. J. Phys. Chem. C.

[26] Xiao G., Man S. (2007). Surface-enhanced Raman scattering of methylene blue adsorbed on cap-shaped silver nanoparticles. Chem. Phys. Lett..

[27] Tong L., Zhu T., Liu Z. (2007). Laser irradiation induced spectral evolution of the surface-enhanced Raman scattering (SERS) of 4-tert-butylbenzylmercaptan on gold nanoparticles assembly. Sci. China Ser. B.

