# Supplementary material for: Simultaneous Thermal Stability and Ultrahigh Sensitivity of Heterojunction SERS Substrates
Source: Nanomaterials (Basel). 2019 May 31;9(6):830. doi: 10.3390/nano9060830 (PMC6631846; doi:10.3390/nano9060830)
Supplement: Supplementary file 1 [file nanomaterials-09-00830-s001.pdf]

Supporting Information

Simultaneous Thermal Stability and Ultrahigh Sensitivity of Heterojunction SERS Substrates

Lingwei Ma [1], Jinke Wang [1], Hanchen Huang [2,*], Zhengjun Zhang [3,*], Xiaogang Li [1] and Yi Fan [4]

[1] Institute of Advanced Materials & Technology, University of Science and Technology Beijing, Beijing 100083, China; mlw1215@ustb.edu.cn (L.M.); wjkgege@126.com (J.W.); lixiaogang@ustb.edu.cn (X.L.)
[2] College of Engineering, University of North Texas, Denton, TX 76207, USA
[3] Key Laboratory of Advanced Materials, School of Materials Science and Engineering, Tsinghua University, Beijing 100084, China
[4] Jiangsu Key Laboratory for Premium Steel Material, Nanjing Iron and Steel Co., Ltd., Nanjing 210035, China; fanyi@njsteel.com.cn
* Correspondence: Hanchen.Huang@unt.edu (H.H.); zjzhang@tsinghua.edu.cn (Z.Z.)

The thickness optimization of Al_2O_3 layer on top of AgNRs

Our choice of Al_2O_3 thickness is based on the following two considerations. First, Al_2O_3 thickness needs to be sufficiently small to ovoid the excessive coverage of Ag surfaces. Second, it needs to be large enough to maximize the thermal stability. With these two considerations, Al_2O_3 layers of 6, 8, 10, 12, 15 and 20 nm were deposited over AgNRs, and these hybrid substrates are denoted as AgNRs-6Al_2O_3, AgNRs-8Al_2O_3, AgNRs-10Al_2O_3, AgNRs-12Al_2O_3, AgNRs-15Al_2O_3 and AgNRs-20Al_2O_3, respectively. Figures S1a-S1b show typical SEM micrographs of AgNRs and AgNRs-10Al_2O_3, separately. Both substrates possessed tilted and closely arranged nanorod morphologies with sharp corners and edges. In view of the ultrathin feature of Al_2O_3 capping, it is hard to be differentiated from the SEM resolution. To this end, HRTEM analysis was adopted to offer visual evidences of the Al_2O_3 capping. Figures S1c-S1e display representative HRTEM images of AgNRs capped with Al_2O_3 layers of 8, 10 and 20 nm, respectively. As can be seen, these Al_2O_3 layers with diverse thickness were only located at the top surfaces of AgNRs, and the side surfaces were largely exposed, which were essential to directly adsorb molecules for SERS amplification.

We then assessed the SERS efficiency of various AgNRs-Al_2O_3 substrates. Figure S2a presents the SERS spectra of 1×10^{-6} M MB on AgNRs-Al_2O_3 substrates with different capping thickness. As expect, in light of the enhancive separation between Ag tips and adsorbates introduced by Al_2O_3 capping, MB Raman signals decreased monotonously with the increase of Al_2O_3 thickness. Herein, the 1622 cm^{-1} Raman peak with strong intensity was chosen to quantify the oxide capping effect on SERS activity. For each sample, the peak value was normalized to that on bare AgNRs to facilitate comparison (see Figure S2b). To be specific, the SERS intensities of MB on AgNRs-Al_2O_3 substrates with 6 nm to 12 nm capping occupied approximately 80% to 70% relative to those on bare AgNRs, and declined moderately to 62% and 56% with further Al_2O_3 deposition to 15 nm and 20 nm, separately. Even though there was some signal degradation after Al_2O_3 capping, these hybrid substrates still exhibited satisfactory SERS enhancement, which was owing to the exposed side surfaces of AgNRs that could directly interact with target molecules.

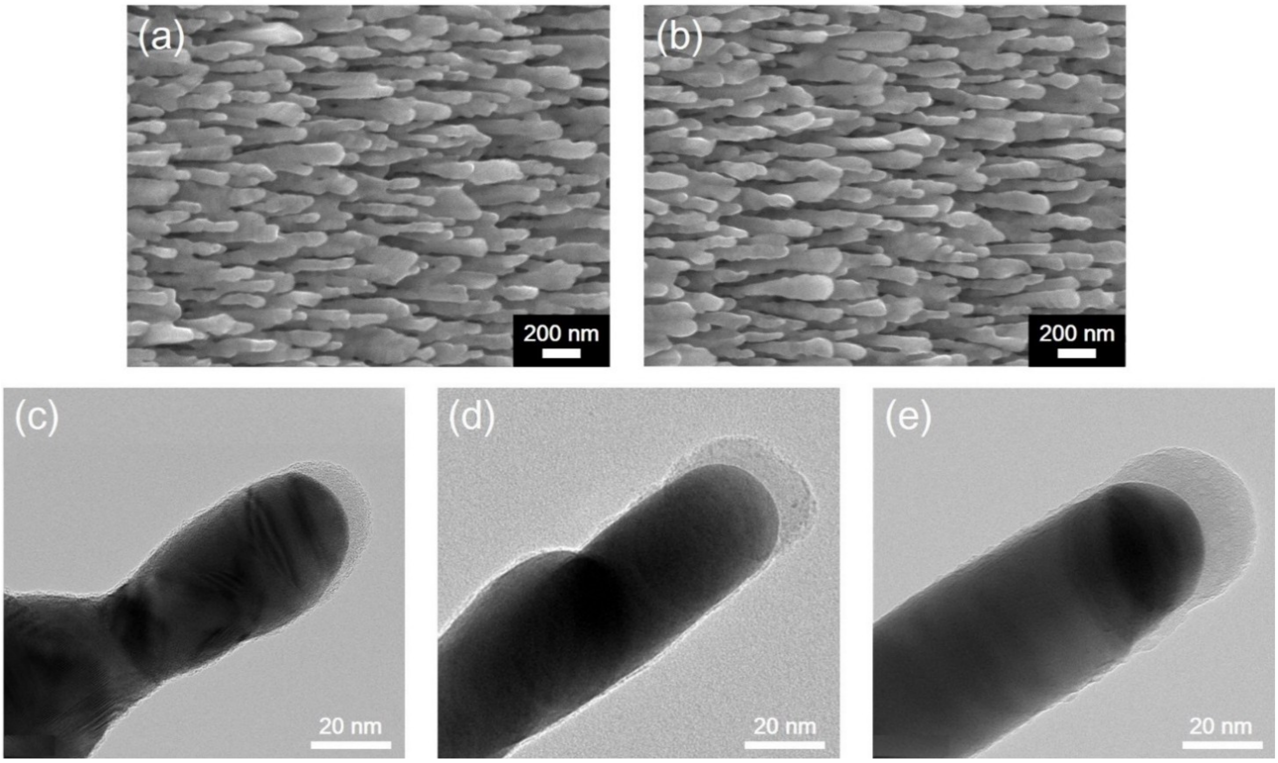

Figure S1. SEM images of (a) uncapped AgNRs and (b) AgNRs-10Al_2O_3 substrates; HRTEM images of AgNRs capped with Al_2O_3 layers of (c) 8, (d) 10 and (e) 20 nm, respectively.

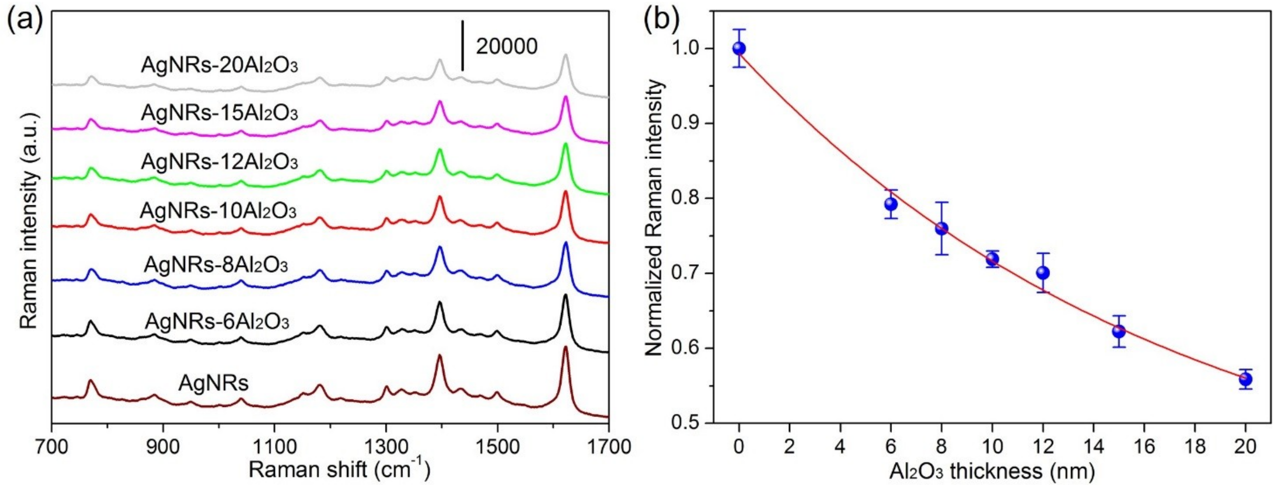

Figure S2. (a) SERS spectra of 1×10^{-6} M MB adsorbed on bare AgNRs and on AgNRs-Al_2O_3 substrates with different capping thickness; (b) The normalized intensities of 1622 cm^{-1} Raman peak of 1×10^{-6} M MB molecules as a function of the Al_2O_3 thickness of different AgNRs-Al_2O_3 substrates.

In spite of the slight decay of SERS signals on AgNRs after Al_2O_3 deposition, the capping layers we introduced here were mainly applied to enhance the corresponding thermal stability. It is known that the high-

temperature robustness of nanostructures relies on their resistance to coarsening. If the molecule diffusion of metal surfaces can be restricted, nanostructures might be stabilized at elevated temperatures. Figure S3 illustrates the morphologies of AgNRs and diverse AgNRs-Al_2O_3 substrates after annealing at 150 °C and 200 °C for 15 min. Evidently, the uncapped AgNRs fused seriously into irregular aggregations after heating, which were not applicable for high-temperature detections. On the other hand, this situation was sharply relieved for AgNRs-Al_2O_3 samples. Specifically, even if the Al_2O_3 coating was only 8 nm for AgNRs-8Al_2O_3, it could prevent the surface diffusion of AgNRs from tips to sides, and thereby the substrate maintained its shape with only small deformation at 150 °C. At 200 °C, the film collapsed to some extent while there were still recognizable nanorod shapes. Subsequently, by depositing 10 nm and 20 nm Al_2O_3 onto AgNRs, the substrates exhibited no discernible morphology variation at 150 °C and coarsened slightly at 200 °C. Therefore, we could maximize the resistance to surface diffusion by capping the top surfaces of AgNRs with Al_2O_3, and enhance the thermal stability of AgNRs-Al_2O_3 films accordingly.

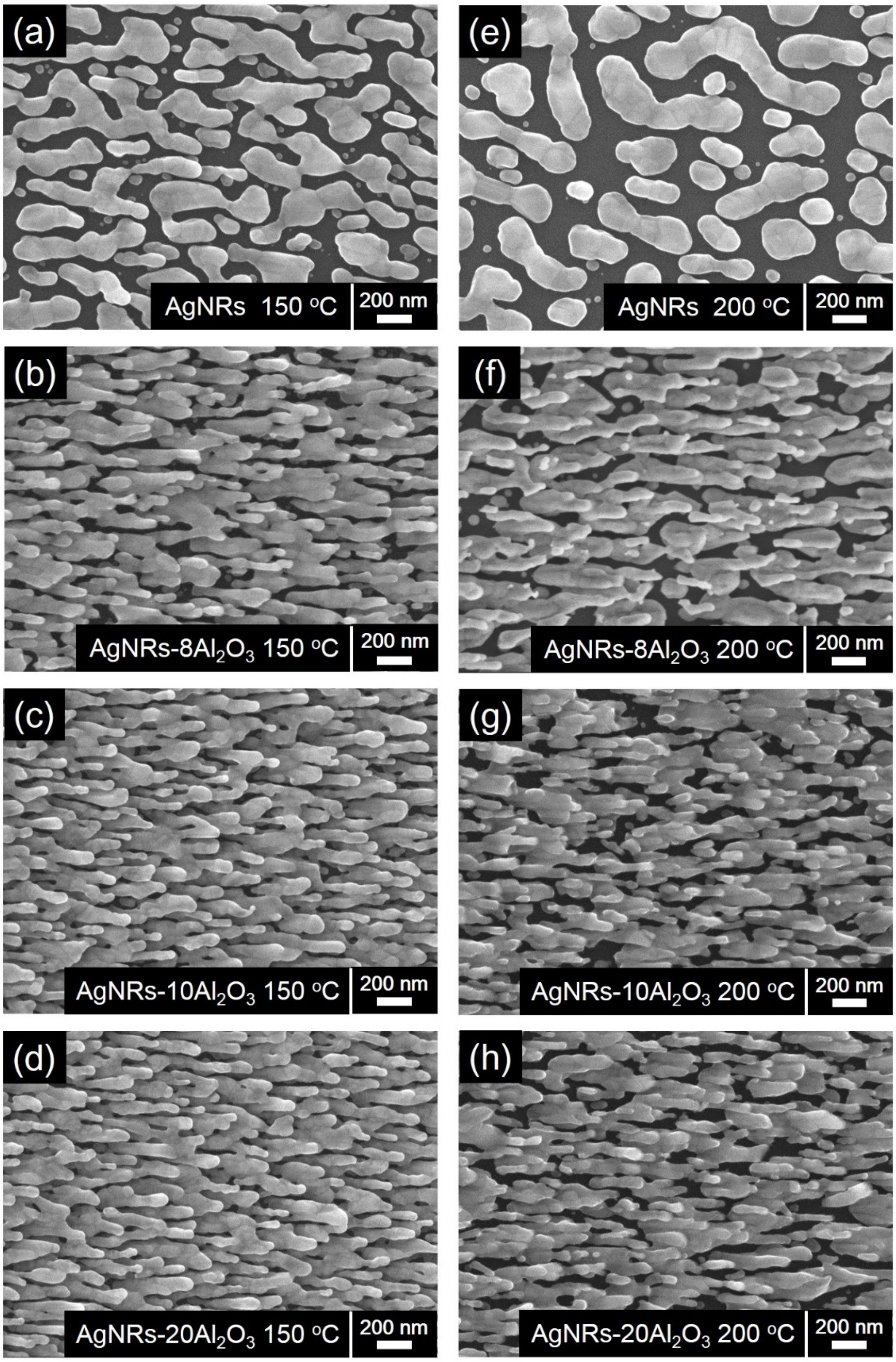

Figure S3. SEM images of AgNRs and different AgNRs-Al_2O_3 substrates after annealing at 150 °C and 200 °C for 15 min.

To characterize *in situ* the melting procedures and structural changes of AgNRs-Al_2O_3 substrates, we measured the reflectivity changes of SERS substrates upon annealing. Figure S4 thoroughly analyzes the

melting behaviors of different AgNRs-Al_2O_3 substrates from 100 °C to 250 °C. For AgNRs-6Al_2O_3 and AgNRs-8Al_2O_3, their reflectivity began to change at ~125 °C and varied sharply at ~180 °C, which might due to the collapse of nanorods. As for the hybrid substrates capped with thicker Al_2O_3 layers of 10-20 nm, their morphology maintained well to ~200 °C. As a result, a 10 nm Al_2O_3 protection layer could restrict the morphological changes of AgNRs at temperatures as high as 200 °C, and AgNRs-10Al_2O_3 possessed minimal degradation in SERS enhancement than the hybrids capped with thicker Al_2O_3 layers. We hence recommend AgNRs-10Al_2O_3 film in this study to guarantee both the stability and sensitivity characters of SERS substrates.

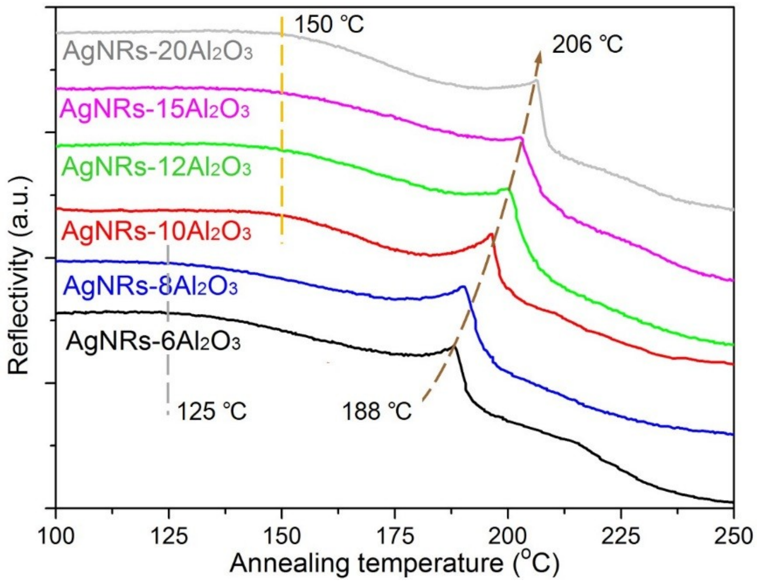

Figure S4. The reflectivity changes of different AgNRs-Al_2O_3 substrates upon annealing from 100 °C to 250 °C.

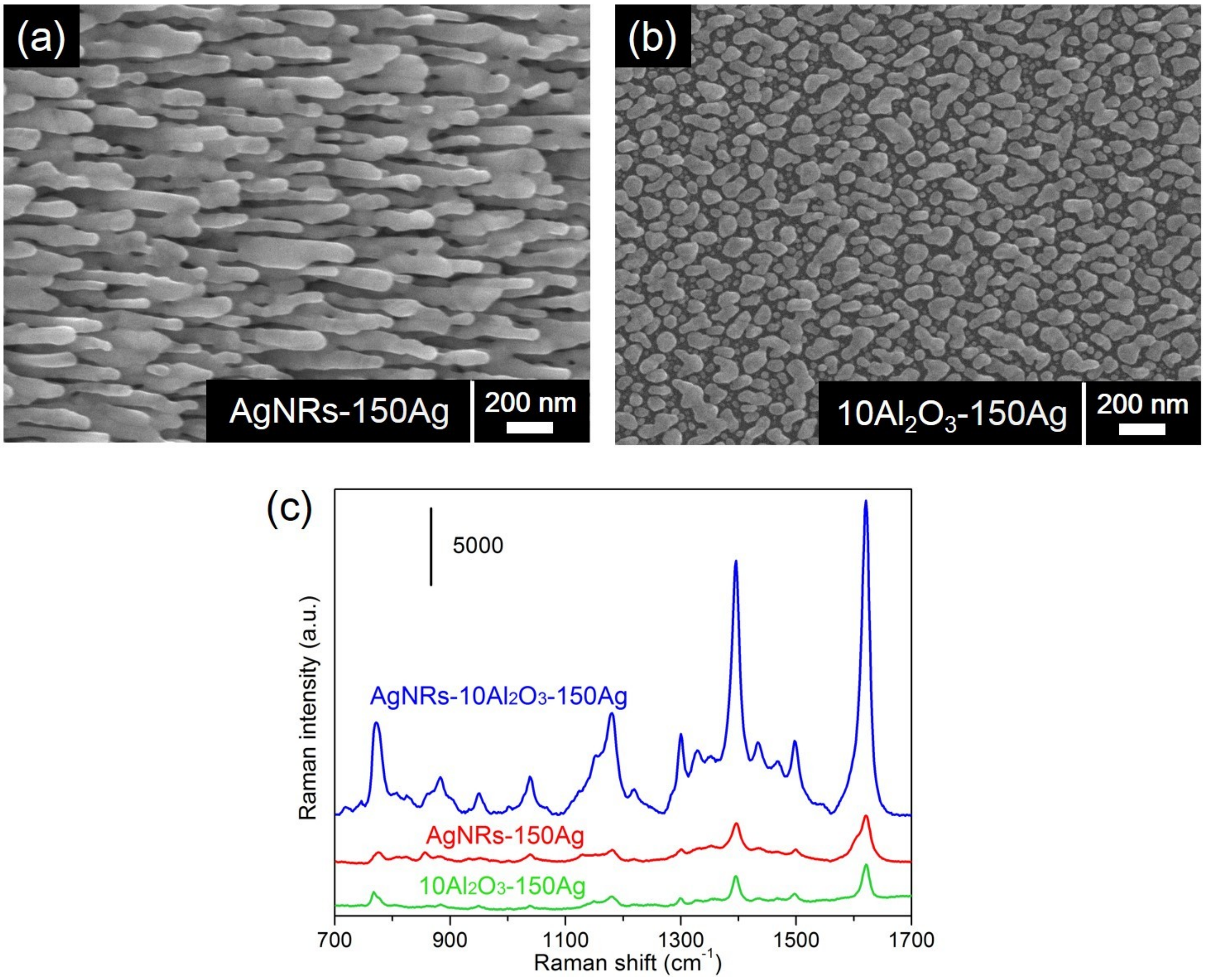

Figure S5. SEM images of (a) AgNRs-150Ag and (b) 10Al_2O_3-150Ag substrates; (c) SERS spectra of 1×10^{-6} M MB molecules adsorbed on AgNRs-10Al_2O_3-150Ag, AgNRs-150Ag and 10Al_2O_3-150Ag substrates, separately.

The calculation of SERS enhancement factor

Methylene blue (MB) molecules were used to calculate the SERS enhancement factor (EF) of AgNRs-Al_2O_3-Ag arrays with 150 nm additional Ag. The SERS spectra of 1×10^{-6} to 1×10^{-10} M MB molecules were first collected on the substrates, as shown in Figure 3a and Figure 4a. Figure 4b plots the Raman peak signals at 1622 cm^{-1} against MB concentrations, which increased gradually and became saturated.

The SERS spectrum of 1×10^{-6} M MB on SERS substrate and the Raman spectrum of 0.1 M MB in solution were used to calculate the SERS EF of AgNRs-Al_2O_3-Ag arrays with 150 nm additional Ag. The EF was calculated by [1, 2]:

$$EF = \frac{I_{SERS}}{I_{Ref}} * \frac{N_{Ref}}{N_{SERS}}$$

In this formula, I_{SERS} is the enhanced intensity of the adsorbed MB molecules on AgNRs-Al_2O_3-Ag arrays, and I_{Ref} is the integrated spontaneous Raman scattering intensity collected from the MB molecules in solution. N_{SERS} is the number of the molecules covering the SERS substrate under the laser spot, and N_{Ref} is the number of MB molecules in aqueous solution excited by the laser. The strong Raman peak intensity at 1622 cm^{-1} was used for EF calculation.

For the Raman equipment in this study, the laser light is focused onto a region with a diameter of 80 μm. To calculate the value of N_{SERS}, MB molecules were assumed to be deposited onto the substrate under the ideal condition that they were absorbed on the substrate surface as a monolayer. With the calculated area of a single MB molecule of 1.29×10^{-18} m^2 [3], the value of N_{SERS} under the laser excitation is approximately 3.9×10^9. The focal volume of the Raman spectrometer is about 5.0×10^6 μm^3. Correspondingly, the N_{Ref} is calculated to be 3.0×10^{15} by multiplying volume, concentration of MB solution and an Avogadro constant (N_A=6.02×10^{23} mol^{-1}). The 1622 cm^{-1} intensity of 1×10^{-6} M MB on AgNRs is about 22350 counts, and that of 0.1 M MB in solution is 187 counts, providing an EF of approximately 1.3×10^8.

References:

[1] S. Xu, Y. Zhang , Y. Luo , S. Wang , H. Ding , J. Xu and G. Li, Ag-decorated TiO_2 nanograss for 3D SERS-active substrate with visible light self-cleaning and reactivation, Analyst 138 (2013) 4519.

[2] J. Yin, Y. Zang, C. Yue, X. He, H. Yang, D. Wu, M. Wu, J. Kang, Z. Wu, J. Li, Multiple coupling in plasmonic metal/dielectric hollow nanocavity arrays for highly sensitive detection, Nanascale 7 (2015) 13495.

[3] E. El Qada, S. Allen, G. Walker, Adsorption of Methylene Blue onto activated carbon produced from steam activated bituminous coal: A study of equilibrium adsorption isotherm, Chem. Eng. J. 124 (2006) 103-110.
